# Male Breast: A Review of the Literature and Current State of the Art of Diagnostic Imaging Work-Up

**DOI:** 10.3390/diagnostics13243620

**Published:** 2023-12-07

**Authors:** Anna D’Angelo, Antonio Portaluri, Flavia Caprini, Carmelo Sofia, Francesca Ferrara, Elvira Condorelli, Ludovica Iaccarino, Francesca Catanzariti, Matteo Mancino, Charlotte M. L. Trombadori, Paolo Belli, Maria Adele Marino

**Affiliations:** 1 Department of Diagnostic Imaging, Oncological Radiotherapy and Haematology, Fondazione Policlinico Universitario A. Gemelli IRCCS, 00168 Rome, Italy; capriniflavia@gmail.com (F.C.); francescaferrara26@gmail.com (F.F.); ludovicaiaccarino@gmail.com (L.I.); matteomancino@gmail.com (M.M.); charlottemargueritelucille.trombadori@guest.policlinicogemelli.it (C.M.L.T.); paolo.belli@policlinicogemelli.it (P.B.); 2 Department of Biomedical Sciences and Morphologic and Functional Imaging, AOU G. Martino, University of Messina, 98100 Messina, Italy; antonio_3_ap@libero.it (A.P.); carm.sofia@tiscali.it (C.S.); condorelli.elvira@alice.it (E.C.); fracatanzariti@hotmail.it (F.C.); marmarino@unime.it (M.A.M.)

**Keywords:** male breast cancer, breast cancer screening, high-risk man, follow-up

## Abstract

Pathological conditions affecting the male breast (MB) share some similarities with those found in women, while others are specific to men. The first part of this review provides an overview of MB disorders, exploring the most common types of MB diseases. The second part then emphasizes the state-of-the-art approaches proposed in the literature for screening and follow-up with MB cancer patients, which highlights the importance of tailored strategies for diagnosis, follow-up, and identifying high-risk populations. Considering the increasing attention in recent years on the topic, transgender individuals are also included in this review. Together with the MB, it is an understudied category thus far. This review aims to raise awareness among radiologists that MBs should be approached differently from female breasts, contributing to the advancement of medical knowledge, improving patient outcomes, and promoting early detection of MB disorders. The review also provides an update on breast cancer and screening in the transgender population.

## 1. Introduction

Male breast (MB) is different from the female breast, thus MB disease cannot be managed in the same way as female breast disorders. Diagnostic and therapeutic approaches for male patients have predominantly followed those established for postmenopausal women [1]. However, there is an unmet need to identify optimal diagnostic and therapeutic pathways tailored specifically for MB, rather than simply transposing approaches used in women [2]. Despite the scientific evidence indicating that MB disease is definitively inferior in comparison to that of the female breast, due to the rarity of the MB malignancy and the lack of multicentric prospective studies [3], this review arrives after several papers in the literature have been published on this topic, most of which are cited here. 

After a brief explanation of the MB development, this review discusses the epidemiology of MB disorders, highlighting the rarity of MB cancer (MBC) and the relatively higher prevalence of benign conditions. It then presents a detailed analysis of diagnostic modalities, including mammography, ultrasound, and magnetic resonance imaging (MRI), discussing their advantages, limitations, and effectiveness in differentiating benign from malignant lesions and aiding in the characterization of specific pathologies. The transgender population is included in this review, considering both the transgender man and the transgender woman, encompassing the definition of terminology and the most up-to-date data on breast cancer incidence and screening within this population.

By examining the existing literature, incorporating available evidence, and considering the unique characteristics of the MB, this review aims to identify and recommend tailored diagnostic and therapeutic pathways for MBC and the high-risk population.

This review does not presume to bridge the existing knowledge gap in MB; rather, it is an effort to bring the reader into the MB world, starting with an overview of benign and malignant disorders and continuing with the newest evidence-based guidelines.

## 2. Male Breast Development

**Figure 1:**
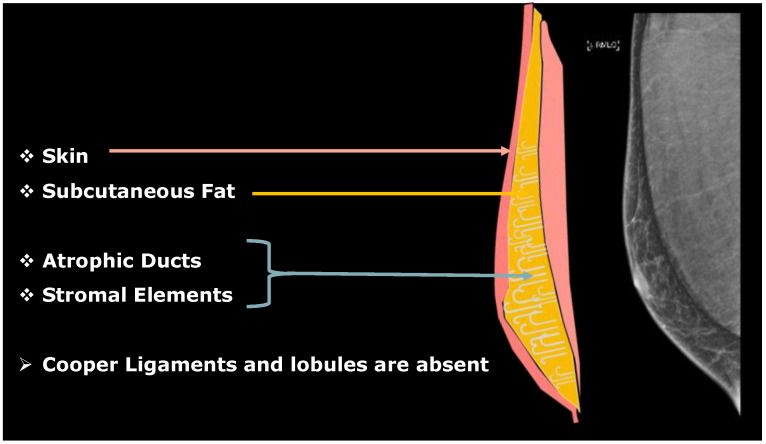
Anatomy of male breast and mammographic appearance.

Male and female mammary glands exhibit significant differences in adulthood, serving distinct functions. At birth, both male and female breasts are comparable [4,5]. However, peripubertal hormonal changes lead to anatomical and functional differences between the genders. Estrogen secretion in girls during puberty results in ductal development, while progesterone contributes to the growth of terminal ductal-lobular units [4]. In contrast, the rise in testosterone levels in boys causes significant involution and atrophy of the ducts, resulting in the adult MB primarily consisting of skin, subcutaneous adipose tissue, stromal elements, and sparse atrophic and blind ducts, with lack of Cooper ligaments [4] (Figure 1). 

## 3. Male Breast Disorders

### 3.1. Benign Breast Disease

MB disorders are relatively uncommon compared to their female counterparts, with a much lower incidence of both benign and malignant conditions [6]. The pathologic conditions affecting the MB differ from those observed in women [7]. Invasive lobular carcinoma is rare in males, as the high levels of testosterone during puberty cause ductal involution and atrophy, inhibiting lobular development [4,5]. However, benign and malignant conditions affecting other anatomical components, including the stroma, fat tissue, and atrophic ducts, are more common [5]. MBC represents less than 1% of all breast cancer cases, but its incidence has been gradually increasing over the years [8]. The average age at diagnosis for MBC is higher than that for female breast cancer, typically occurring between the ages of 60 and 70 [8]. However, certain risk factors such as family history, BRCA2 mutations, and hormonal imbalances can predispose some men to a higher risk of developing breast cancer. The following sections will discuss both benign and malignant conditions affecting the MB. Due to the differences in breast structure between men and women, certain commonly seen benign lesions in women, such as fibroadenomas and cysts, are not typically expected in men due to the absence of lobules [9]. However, isolated cases of fibroadenomas in MB have been reported [10]. Fibroadenomas are recognized to have both estrogen and progesterone receptors, and the majority of documented cases of male fibroadenomas have been observed in MTF transgender patients or individuals undergoing estrogen therapy for medical reasons, such as prostate carcinoma [11]. Table 1 lists a comprehensive range of benign breast lesions that may potentially affect men. 

Gynecomastia is the most prevalent benign disorder and affects approximately 57% of men above 40 years old. It is characterized by a non-neoplastic enlargement of the MB due to a benign proliferation of the ductal and stromal components. Multiple factors contribute to its development, as summarized in Table 2 [4]. 

Clinically, gynecomastia presents as a subareolar palpable mass, often mobile and tender, or as a peri-areolar burning sensation; this is the most prevalent condition for males presenting with breast complaints [12]. In the case of diagnosis of gynecomastia at physical examination, imaging is not appropriate [5]. 

**Figure 2:**
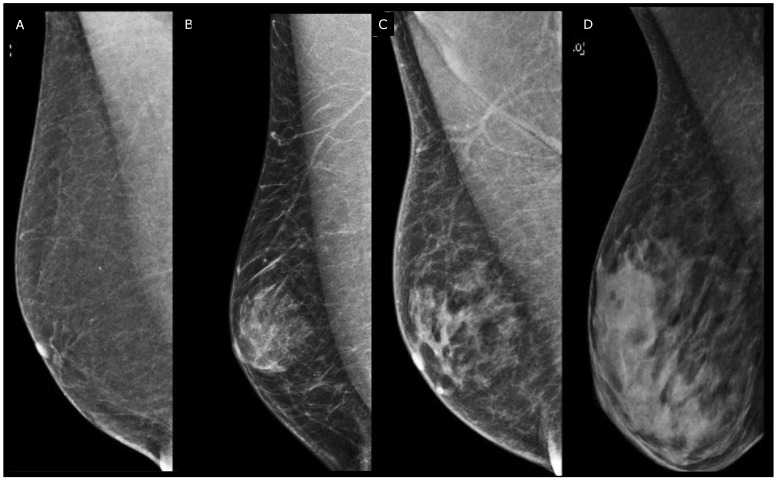
Right medio-lateral-oblique digital mammography of different types of male breast. (**A**) Pseudo-gynecomastia: the breast is almost entirely fatty. (**B**) Nodular gynecomastia: in the retro-areolar region, there is a nodular density that blends into the surrounding subcutaneous fat, resulting in indistinct border. (**C**) Dendritic gynecomastia: there are fibrous extensions of dendritic gynecomastia (flame shaped) in the breast. (**D**) Diffuse gynecomastia: heterogeneously dense breasts consisting of both nodular and dendritic components that closely resemble female breasts in a transgender patient receiving high-dose estrogen therapy.

The mammographic assessment reveals three distinct patterns: nodular, dendritic, and diffuse [13]. Nodular gynecomastia is an early and reversible manifestation, appearing as a retro-areolar fan-shaped density with indistinct borders, representing hyperplasia of intraductal epithelium and stromal edema. Dendritic gynecomastia indicates the fibrotic phase, characterized by dilated ducts and hyalinized stromal fibrosis. Mammographic features include a flame-shaped density with linear finger-like projections into the subareolar fatty tissue. Diffuse gynecomastia results from chronic estrogen exposure and can exhibit features of both nodular and dendritic forms or resemble the architecture of the female breast [5]. Pseudo-gynecomastia, primarily observed in overweight or obese individuals, results from excessive fatty tissue stimulation without fibro-glandular components and is a key differential diagnosis to consider (Figure 2). 

**Figure 3:**
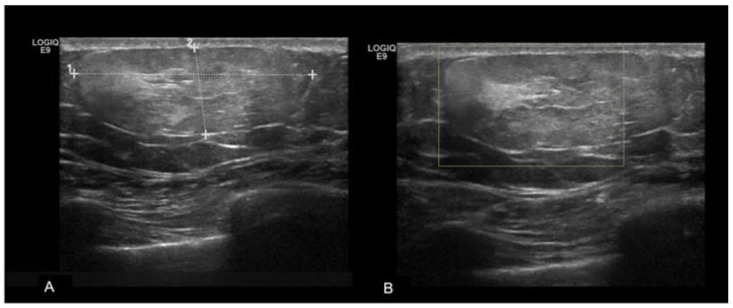
A 35-year-old men with a palpable lump in the inner quadrants of the left breast. On a B-mode (**A**) breast ultrasound, there is an oval shaped and hyperechoic mass, with circumscribed margins and parallel orientation. No posterior feature is associated. On color-doppler (**B**), there are no signs of vascularization. The imaging features are consistent with a lipoma.

Lipomas, the second most common benign lesions in the MB, are composed of adipose tissue and typically present as soft, mobile, and palpable masses in clinical examination. However, they are frequently detected incidentally. Mammographically, lipomas appear as well-defined radiolucent oval masses with a radiopaque capsule that might be difficult to detect [14,15]. On ultrasound, lipomas demonstrate an oval shape, parallel orientation, iso-hyperechoic echogenicity, and lack detectable vascular flow [16] (Figure 3). 

**Figure 4:**
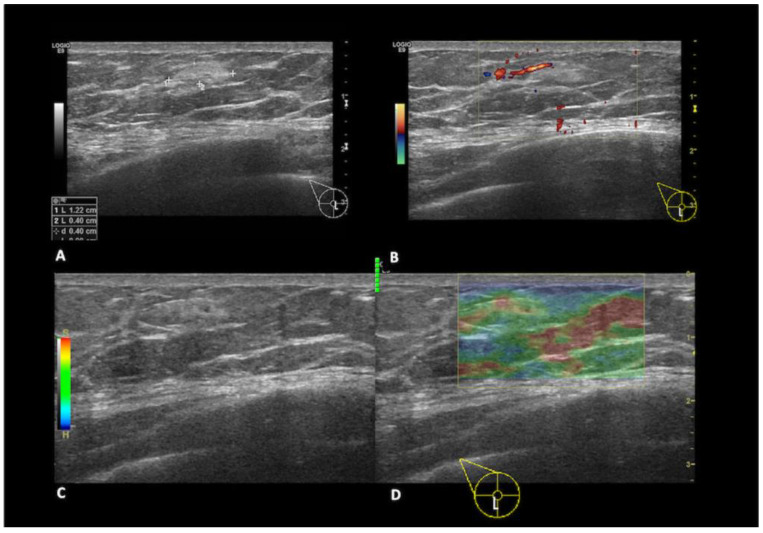
A 49year-old patient with a history of a blunt trauma on the right breast. In the sub-areolar region (**A**) of the right breast, there is a hyperechoic mass with anechoic internal components and (**B**) no signs of vascularization at color-doppler. (**C**,**D**) Strain elastography evaluation shows that the mass has an intermediate elasticity. The findings are consistent with fat necrosis.

Epidermal cysts, also referred to as sebaceous cysts, represent the third most prevalent benign lesions in the MB. They arise from occluded hair follicles and appear as well-defined oval masses beneath the skin on mammography. Ultrasound examination reveals a homogeneous or heterogeneous hypoechoic oval lesion with no detectable vascular flow. The diagnosis is highly likely if the lesion exhibits a connecting part with the skin [17]. Fat necrosis is a benign condition often resulting from trauma, biopsy, or surgery (Figure 4). Various imaging findings can be attributed to fat necrosis depending on the stage of necrosis [5]. Mammographically, it can manifest as an oil cyst, presenting as a round, circumscribed mass with a thin calcified rim. In some cases, it may appear as an oval mass associated with microcalcifications or coarse calcifications, posing a challenge in ruling out a malignancy. Sonographic features of fat necrosis vary, ranging from echogenic mobile internal bands to solid masses or complex masses with nodules [18].

**Figure 5:**
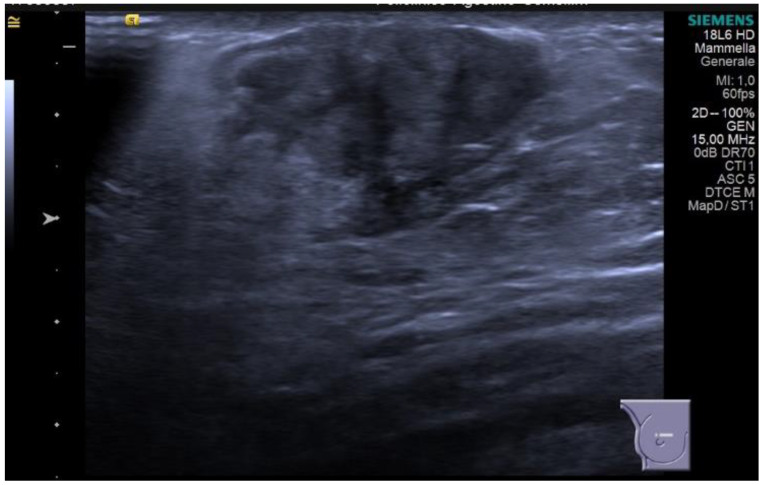
Palpable lump on the right side in a 71-year-old man, with a personal history of type 1 diabetes and hypertension. On breast ultrasound, there is a subareolar irregular shaped and hypoechoic mass, with not-circumscribed margins and vertical orientation. No posterior feature is associated. The mass is suspicious for malignancy (BI-RADS 4b) and a core-needle biopsy was performed. Final histology: fibrosis, gynecomastia, and chronic inflammation are consistent with diabetic mastopathy.

Diabetic mastopathy, although rare in men, is strongly associated with type 1 diabetes. It may present as single or multiple lesions in the breast, and imaging findings can mimic malignancy. Mammographically, it can appear as an ill-defined solid mass, architectural distortion, or asymmetric densities. Ultrasonography reveals an irregular hypoechoic mass with posterior acoustic shadowing and internal vascularization (Figure 5). 

However, in some cases, diabetic mastopathy may appear as well-circumscribed masses without associated posterior features or vascularization, suggesting a benign nature [18,19]. 

**Figure 6:**
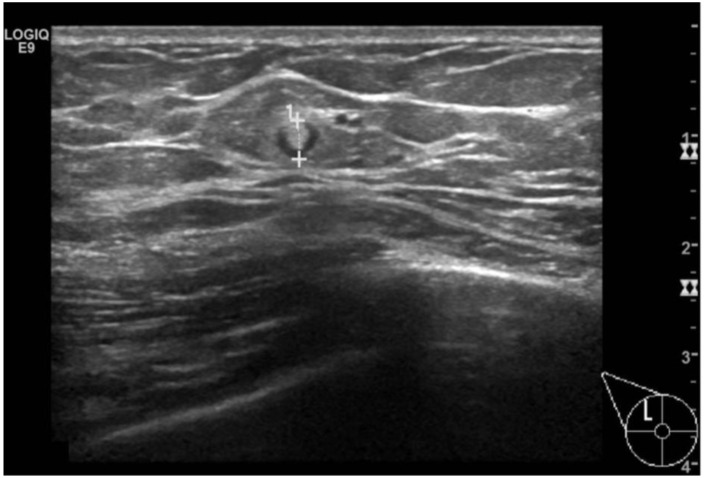
An intra-mammary lymph-node in the upper-outer quadrant of the right breast of 70-year-old men. B-mode ultrasound shows the small reniform mass with a central hyperechoic fatty hilum and the hypoechoic cortex.

Abscesses in the MB are rare conditions, mostly located in the subareolar region and resulting from traumas that create a pathway for microorganisms, such as nipple piercing [20]. Staphylococcus aureus and S. epidermidis are commonly associated with MB abscesses. Clinically, abscesses present as nodular, erythematous areas, and fever. Mammography findings may include breast enlargement, skin thickening, or irregular masses with or without calcifications, posing challenges in differentiating them from malignancies. Ultrasound reveals irregular complex masses with surrounding fat tissue hyper-echogenicity and vascularization at the rim [21]. Intramammary lymph nodes are frequently encountered in female breast screening examination, as opposed to MB (Figure 6). Mammographically, they appear as sub-centimeter, oval, or reniform masses with radiolucent fatty hilum. Ultrasonography shows echogenic cortices with central hyperechoic fatty hilum [22]. Pseudo-angiomatous stromal hyperplasia (PASH) is a rare hormone-dependent condition that often underlies gynecomastia. It can mimic fibroadenomas on conventional imaging, presenting as single, ill-defined elastic masses ranging from 3 mm to 18 cm [23].

### 3.2. Malignant Breast Disease

MBC is a rare disease, comprising only 0.17% of all male cancers [24]. It is associated with a poorer prognosis compared to breast cancer in females [2,25]. Late-stage diagnosis contributes to this poor prognosis [25], primarily due to limited public awareness, resulting in patients seeking medical attention when symptoms are severe and the disease is advanced. Moreover, the stigma associated with MBC being perceived as a “woman’s disease” often causes of delayed physician referral [26]. MBC is characterized by high expression of hormone receptors, estrogen receptors (ERs), and progesterone receptors (PRs), along with low expression of human epidermal growth factor receptor 2 (HER2) [5]. The average age at diagnosis is 59 years, which is higher than that of female breast cancer [25]. 

The histological subtypes of MBC are diverse, and include invasive ductal carcinoma, invasive lobular carcinoma, ductal carcinoma in situ, and papillary carcinoma, among others (Table 3).

**Figure 7:**
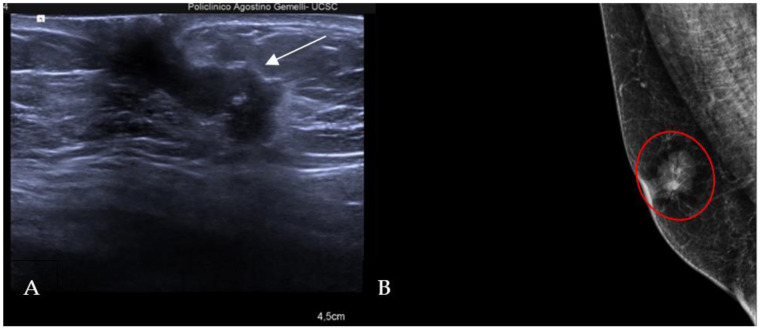
A 60-year-old man with gynecomastia presenting a retroareolar lump on the right breast with nipple retraction and without nipple discharge. Histological examination revealed an invasive ductal carcinoma (ER + 90%, PR + 60%, HER2 score 1+). Ultrasound examination (**A**) shows a hypoechoic irregular mass (white arrow) in the retroareolar region, with lobulated margins. A mammography (**B**) shows an irregular hyperdense retroareolar mass with lobulated margins (red circle).

The most common histological subtype of primary malignant breast lesions in men is invasive ductal carcinoma (IDC) [27,28], shown in Figure 7.

**Figure 8:**
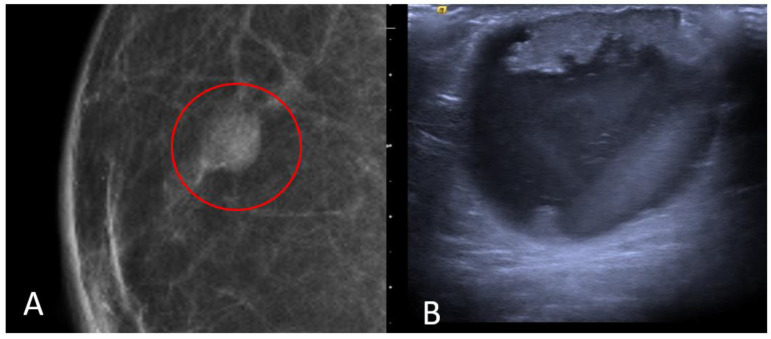
Invasive papillary carcinoma in a 59-year-old man with bloody nipple discharge. Magnification of mediolateral oblique view of the right breast (**A**) shows a focal high-density mass with well-circumscribed margins (red circle). Ultrasound (**B**) shows a complex cyst with mixed solid and cystic morphologic features.

IDC typically originates from the terminal duct–lobular unit [28,29]. Clinical features include a palpable unilateral retroareolar mass with nipple retraction and skin thickening [28,29]. Around 25% of cases may present with bloody nipple discharge [28]. IDC can be associated with ductal in situ components in up to 50% of cases [30]. Mammographically, IDC appears as a retroareolar irregular high-density mass with spiculated or micro-lobulated margins [30,31]. Unlike in women, IDC in men is rarely associated with microcalcifications due to the involution of the ductal system caused by the absence of estrogen and progesterone stimulation [28,29]. Ultrasonography reveals solid, hypoechoic, and irregular masses with margins ranging from microlobulated to spiculated [32]. Papillary carcinoma (PC) is the second most common invasive subtype of MBC and has a higher incidence in men compared to women [33]. PC is characterized by neoplastic proliferation of cells with fibrovascular stalks lacking an intact myoepithelial cell layer [34]. It typically presents with bloody nipple discharge and occurs in the subareolar region. Mammographically, PC may exhibit well-circumscribed or spiculated margins, while ultrasound imaging may reveal a dilated duct or cyst, often appearing as a complex cyst with solid papillary projections along the cyst wall [35,36] (Figure 8).

Invasive lobular carcinoma (ILC) is rare in men due to the lack of terminal lobules in MB architecture, which is linked to low estrogen exposure during puberty [29]. Studies report a 2.6% to 1.5% incidence of lobular carcinoma in men [33,37]. There is a potential association between lobular carcinoma and Klinefelter syndrome, attributed to increased estrogen levels [38]. Mammographic features may include a spiculated mass or architectural distortion, while ultrasound imaging may show an irregular hypoechoic mass or, less commonly, be sonographically occult [39]. 

Secondary metastatic involvement of the breast should be taken into consideration when evaluating uncommon breast masses, particularly in individuals with a history of primary cancer. Melanoma is the most frequent primary tumor in this scenario, followed by lymphoma, lung cancer, and prostate cancer [40] (Table 4). 

The incidence of secondary breast metastases is relatively low, accounting for only 2–5% of all malignant breast tumors [37,38]. Clinical and radiological features of secondary breast metastases often resemble those of primary breast cancer, posing a challenge for definitive diagnosis. Imaging characteristics may include multiple and bilateral well-circumscribed or spiculated breast masses, with or without associated calcifications [29]. Nonetheless, the presence of a known primary malignancy, along with the clinical context and radiological findings, may raise suspicion of metastatic involvement [39]. In such cases, biopsy or histological examination of the tissue is necessary to confirm the diagnosis, as the treatment approach may differ from that of primary breast cancer [41]. In conclusion, the possibility of metastatic disease should be considered in the differential diagnosis of breast masses, especially in patients with a history of cancer, as it can significantly influence patient management and prognosis. According to the American College of Radiology [42], mammography digital breast tomosynthesis and ultrasound are appropriate depending on patient’s age and physical examination. MRI is not routinely recommended and might be not appropriate for MB (Table 5).

## 4. High-Risk Screening

Screening examinations for BC in women are an essential component of overall population health, aiming to detect and timely address breast cancer regardless of individual risk factors or personal history. In women, overdiagnosis in breast cancer screening programs is widely debated [43] while in men we rather face underdiagnosis [41], with a significant disparity in mortality rates compared to women. While the mortality for female BC has decreased over the years, the mortality rate for MBC has not followed the same trend [41]. Due to the low incidence of MBC, routine BC screening is not typically performed in men as it is not cost-effective. However, focusing on high-risk individuals may offer a potential strategy to reduce mortality in MBC. The identification of high-risk men has improved with the wider availability of genetic testing and counseling. Certain genetic mutations, such as BRCA1 and BRCA2, significantly increase the risk of BC in men. Other less common genes, including PTEN, CHEK2, and BARD1, have also been identified in the high-risk population. In addition to genetic mutations, personal or family history of BC, ethnicity, and multiple risk factors contribute to the increased risk of developing BC in men. Early detection plays a crucial role in improving survival rates for various types of cancers, underscoring the urgency to develop new strategies for MBC. Although limited by selection bias and retrospective design, several studies have explored the feasibility of screening programs for high-risk men, yielding promising results. Mammography has shown high sensitivity and negative predictive value in both symptomatic and asymptomatic men. Moreover, studies suggest that screening mammography could be valuable for men at increased risk for BC, reporting a similar cancer detection rate as in average-risk women. The National Comprehensive Cancer Network (NCCN) now recommends annual mammogram screening for men with BRCA1/2 mutations and gynecomastia starting at age 50 or 10 years before the earliest known BC in the family [44]. Additionally, clinical breast examination and training in breast self-examination are advised starting at 35 years of age, along with prostate cancer screening starting at 40 years old. The role of MRI in screening and as an initial imaging modality is not indicated in any clinical scenario at present. Ultrasound is still a topic of debate and is not recommended as a screening tool but rather as an adjunct to mammography or digital breast tomosynthesis for patients with suspicious findings. Screening recommendations for high-risk men are summarized in Table 6.

## 5. Follow-Up

MBC survivors face an increased risk of developing second primary cancers, such as pancreatic cancer and colorectal cancer, particularly in those with pathogenic BRCA1, BRCA2, PALB2, and CHEK2 variants. The American Society of Clinical Oncology (ASCO) recommends [45] annual mammography in cases of lumpectomy and offers contralateral mammography in carriers of genetic mutations. However, the consensus for patients without genetic mutations is yet to be reached. Breast MRI is not routinely recommended for men with a history of BC. Genetic counseling and testing for germline mutations should be offered to male BC patients. Follow-up recommendations for male patients are similar to those for females, including annual bilateral mammography and regular physical examinations for several years after surgery. Follow-up recommendations are summarized in Table 6.

## 6. Transgender Individuals, Breast Cancer and Screening

**Figure 9:**
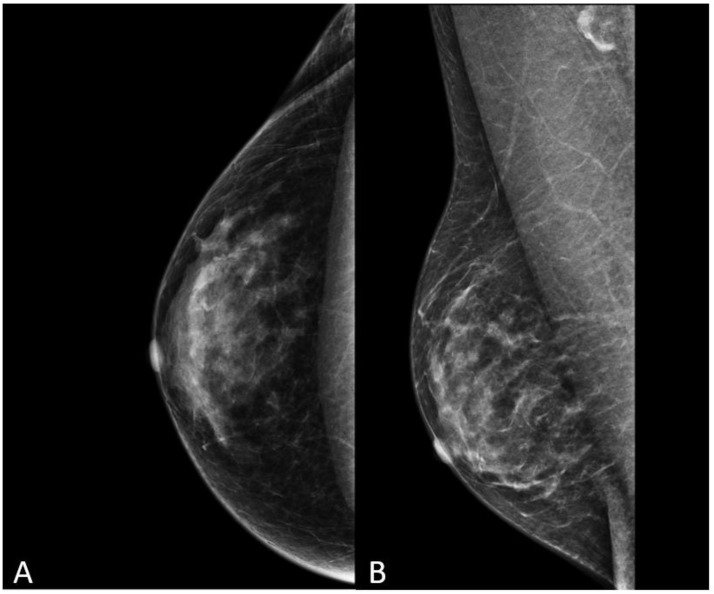
Mammography in cranio-caudal (**A**) and medio-lateral oblique (**B**) views in a 52-year-old transgender woman after 5 years of hormone therapy.

“Transgender” is a comprehensive umbrella term that refers to individuals whose gender identity (their internal perception of gender) and/or gender expression (their outward presentation of gender identity) deviate from the sex assigned to them at birth [46]. Male-to-female (MTF) transition refers to the process in which an individual assigned male at birth undergoes various changes, both social and medical, to align their gender identity with that of a female. Female-to-male (FTM) is the opposite. Transition MTF (Figure 9) may include steps such as feminizing hormone therapy (estrogen with an adjunctive antiandrogen medication (e.g., spironolactone) to suppress endogenous testosterone) to induce physical changes like breast development, as well as gender-affirming surgeries such as breast augmentation and facial feminization surgery or gender confirmation surgery (commonly known as sex reassignment surgery) [47]. The transition FTM can include the gender-affirming surgery top surgery with removal of breast tissue, orchiectomy, hysterectomy with bilateral salpingo-oophorectomy, facial feminization surgery, chondrolaryngoplasty, and gender-affirming hormone therapy: testosterone (by IM injection or by transdermal patch, gel, or implant) [47].

In recent years, there has been a growing emphasis within the scientific community on the lesbian, gay, bisexual, transgender, queer, intersexual, asexual (LGBTQIA+) population, particularly with a focus on transgender and gender-diverse individuals [46]. On a global scale, the proportion of adults identifying as transgender ranges from 0.3% to 0.5%, while approximately 0.5% to 4.5% of adults are estimated to identify as gender-diverse individuals [48]. The healthcare system still grapples with two fundamental challenges: the insufficient training of healthcare professionals on gender incongruence, leading to mistreatment and discrimination, and the absence of consistent, extensive prospective data. 

Scientific evidence regarding the risk of developing breast cancer (BC) in this population and the associated benefits of BC screening remains limited and inadequate. The risk of developing BC tends to increase in transgender women compared to cisgender men (standardized incidence ratio [SIR], 46.7; 95% CI, 27.2–75.4), although it does not reach the level of risk in cisgender women (SIR, 0.3; 95% CI, 0.2–0.4) [49]. Conversely, in transgender men, the risk of BC decreases compared to cisgender women (SIR, 0.2; 95% CI, 0.1–0.5), but it still remains higher than the risk observed in cisgender men (58.9; 95% CI, 18.7–142.2). Regarding the screening programs, transgender and non-conforming gender populations exhibit lower adherence compared to the cisgender population. This is attributed to socio-economic barriers and the absence of well-defined recommendations supported by scientific evidence. The current guidelines from the National Comprehensive Cancer Network (NCCN) [50] and AIOM [51] do not provide specific recommendations for transgender individuals due to the aforementioned dearth of scientific evidence. The American College of Radiology (ACR) [52] governs screening protocols for the transgender population, considering factors such as age, hormone therapy exposure, surgical history, and risk categories.

## 7. Discussion

The initial consideration of this review is that MB disease, which includes benign conditions and BC, remains an area of limited focus and understanding when compared to its female counterpart. The literature review conducted reveals that, in the past ten years, there has been little change in terms of imaging findings for male malignant and benign breast diseases. This lack of progress is primarily due to the absence of significant studies, particularly multicentric and prospective studies, addressing this topic. For instance, there is a lack of evidence regarding the advantages of performing bilateral or unilateral mammograms in males over 25 years of age with an indeterminate palpable mass [41].

While the overall incidence of MBC is lower compared to female BC, the risk is significantly elevated among certain populations, such as those with a family history of breast cancer, BRCA gene mutations, Klinefelter syndrome, and other genetic predispositions. This prompts the question of whether a screening approach for the entire male population is both economically and clinically justified. Instead, focusing on high-risk individuals can optimize resources while enhancing early detection and improving outcomes. Moreover, the lack of appropriate screening and BC awareness in men has resulted in delayed diagnoses and limited treatment options. For these reasons, it is crucial to rectify this by raising awareness about MB disease and dismantling societal stereotypes that hinder open discussions and early interventions. 

Our review underlines as the paucity of international screening programs specifically tailored to high-risk men is a critical issue. This article is intended to draw attention to the urgent need to establish more detailed and unambiguous screening protocols that consider the unique physiological and genetic characteristics of this population. Prioritizing high-risk male individuals also aligns with the concept of precision medicine, wherein screening and diagnostic approaches are tailored to an individual’s genetic makeup, lifestyle, and risk factors. Genetic counseling and testing for mutations such as BRCA1 and BRCA2 can play a pivotal role in identifying individuals who could benefit from more intensive screening. Furthermore, the scarcity of research on high-risk MB disease limits our understanding of its underlying biology and appropriate management. Female BC research has contributed significantly to personalize treatment strategies based on genetic markers, but such advancements have not been paralleled in the male context. Increased research efforts targeting high-risk male populations are essential to unravel the genetic, molecular, and clinical nuances of the disease, enabling the development of tailored therapeutic approaches. Addressing the screening disparities between male and female breast diseases requires a two-pronged approach. Firstly, acknowledging the heterogeneity within the male population and identifying high-risk individuals through genetic counseling and testing can lay the foundation for more effective screening. Secondly, research endeavors focused on high-risk male populations are vital for advancing our knowledge and subsequently improving screening and treatment strategies. 

Transgender individuals constitute a socially disadvantaged group, often subject to discrimination and unequal healthcare, leading to suboptimal cancer outcomes. It remains uncertain whether the use of exogenous testosterone therapy influences the incidence of breast cancer in transgender men. While additional evidence is required to enhance BC screening guidelines for transgender individuals, we suggest that transgender men who have not undergone chest masculinization surgery adhere to the existing breast cancer screening guidelines established for cisgender females.

## 8. Conclusions

In conclusion, MB disorders present unique challenges in diagnosis and management compared to their female counterparts. While male MBC is rare, its incidence is gradually increasing and has a poorer prognosis compared to breast cancer in women, emphasizing the need for improved understanding and tailored approaches for diagnosis and treatment. Mammography remains the standard imaging modality in both symptomatic and asymptomatic patients. Ultrasound, which is still a topic of debate, provides additional information in case of mammographic suspicious findings and to guide biopsy. An annual mammogram in BRCA 1–2 mutation carriers with gynecomastia starting at age 50 is recommended. Current international recommendations do not include breast MRI in male patients. Genetic testing should be considered in cases with a suspected hereditary predisposition. The integration of clinical expertise, radiology, pathology, and oncology through multidisciplinary collaboration is essential to ensure accurate diagnosis, appropriate staging, and tailored treatment plans. 

## 9. Future Directions

More efforts are needed in multicentric, prospective larger and longer-term dedicated studies for MB, especially to create more robust guidelines for screening in high-risk men no-carriers of BRCA 1–2 mutations (including PTEN, CHEK2, and BARD1 gene mutation carriers and personal or family history of BC, etc.). Moreover, a consensus should be reached recommending contralateral annual mammogram for the follow-up in BC patients without genetic mutations. Even for transgender individuals, multicentric and prospective trial are required to delineate the exact risk of BC in this population and to create more robust screening guidelines. Finally, radiologists must be aware of the needs of transgender individuals undergoing breast imaging so that optimal care can be delivered.

## Figures and Tables

**Table 1:** Spectrum of benign lesions, both non-neoplastic and neoplastic, potentially affecting male breast. A list of lesions that are not expected to occur in male breasts is found in the last column.

Benign Non-Neoplastic	Benign Neoplastic	Not Expected
Gynecomastia Pseudogynecomastia Intramammary Lymph Node Sebaceous Cyst Diabetic Mastopathy Hematoma/Fat Necrosis Abscess Venous Malformation Secondary Syphilis Nodular Fasciitis	Angiomyolipoma Schwannoma Myofibroblastoma Intraductal Papilloma Lipoma Pseudoangiomatous stromal hyperplasia	Lactating adenoma Lobular Carcinoma Fibroepithelial Lesions (Fibroadenoma; Phyllodes tumor & choriocarcinoma)

**Table 2:** List of the most common causes of gynecomastia. Hepatocellular carcinoma (HCC).

Common Causes of Gynecomastia	
Physiologic Causes	Neonatal period or infancy, Puberty, Senescence
Drugs	Marijuana, Anabolic Steroids, Leuprolide acetate, Thiazide diuretics, Cimetidine, Tricyclic antidepressants, Estrogen Therapy, Spironolactone, Digitalis
Cirrhosis	
Hypogonadism	Klinefelter syndrome (XXY); Pituitary hormone deficiency;
Neoplasms	Germ cell tumors, Leydig cell tumor, Sertoli cell tumor, Adrenocortical tumors, Pituitary tumors, HCC
Hyperthyroidism	
Chronic Renal Disease and Dialysis	
Idiopathic	

**Table 3:** Spectrum of malignant lesions in male breast.

Malignant Lesions		
Invasive Ductal Carcinoma	Liposarcoma	Basal Cell Carcinoma of the Nipple
Invasive Lobular Carcinoma	Dermatofibrosarcoma Protuberans	Metastases
Papillary Carcinoma	Pleomorphic hyalinizing angiectatic tumor (PHAT)	
Adenoid Cystic Carcinoma	Lymphoma	

**Table 4:** Imaging findings of the most common breast metastases.

Primary Tumor	Imaging Findings
Melanoma	Multiple and bilateral, well-circumscribed or spiculated breast masses with or without calcifications
Lymphoma	Enlarged, hypoechoic, or heterogeneous breast mass
Lung Cancer	Multiple nodules or masses with or without calcifications, spiculated margins, and associated pleural or pulmonary abnormalities
Prostate Cancer	Unilateral or bilateral solid or cystic breast masses, often located near the nipple or areola

**Table 5:** American College of Radiology recommendation. MG= mammography; DBT = digital breast tomosynthesis; US= ultrasound; MRI= magnetic resonance imaging.

Male Patient	Physical Examination	MG	DBT	US	MRI
Any Age	Gynecomastia	Usually Not Appropriate	Usually Not Appropriate	Usually Not Appropriate	Usually Not Appropriate
<25 yo	Indeterminate palpable mass	May be appropriate	May be appropriate	Usually Appropriate	Usually Not Appropriate
≥25 yo	Indeterminate palpable mass	Usually Appropriate	Usually Appropriate	May be appropriate	Usually Not Appropriate
≥25 yo	Indeterminate palpable mass	Suspicious	Suspicious	Usually Appropriate	Usually Not Appropriate
Any Age	Suspicious	Usually Appropriate	Usually Appropriate	Usually Appropriate	Usually Not Appropriate

**Table 6:** Screening and follow-up recommendations for high-risk men. R = recommended; NS = not specified; NR = not recommended; BC = breast cancer.

Group	Conditions	Physical Examination	Genetic Counseling & Genetic Testing	Breast Self-Examination	Annual MG	Breast MRI
NCCN (2021) [20]	BRCA 1/2 mutation carriers	R	NS	R	R	NR
ASCO (2022) [7]	History of BC and BRCA 1/2 mutation carriers	NS	NS	NS	R	NS
	History of BC	NS	R	NS	R Ipsilateral in case of lumpectomy Contralateral in case of mastectomy	NR
AIOM (2021) [27]	History of BC	R	NS	R	R	NR
	BRCA 1/2 mutation carriers	NS	NS	NS	R	NR

## Data Availability

The data presented in this study are available on request from the corresponding author. The data are not publicly available due to privacy restrictions.
